# Roles of Restricted Mean Survival Time and Restricted Mean Time Lost in Evaluating Immune Checkpoint Inhibitor Efficacy for Extensive-Stage Small Cell Lung Cancer

**DOI:** 10.1158/2767-9764.CRC-25-0387

**Published:** 2026-01-12

**Authors:** Seren Durer, Pingfu Fu, Zhengyi Chen, Afshin Dowlati

**Affiliations:** 1 University Hospitals Seidman Cancer Center and Case Western Reserve University, Cleveland, Ohio.; 2 Department of Population and Quantitative Health Sciences, Case Western Reserve University School of Medicine, Cleveland, Ohio.

## Abstract

Chemo-immunotherapy is the current standard of care for extensive-stage small cell lung cancer (ES-SCLC), but interpreting hazard ratios (HR) from Cox models can be challenging when immune checkpoint inhibitors (ICI) produce early crossing or delayed separation of Kaplan–Meier curves. Restricted mean survival time (RMST) and restricted mean time lost (RMTL) have emerged as alternative metrics that do not rely on the proportional hazards (PH) assumption. We conducted a systematic review and meta-analysis using reconstructed individual patient data (IPD) from phase III trials comparing ICIs to standard chemotherapy in the first-line setting for ES-SCLC. Kaplan–Meier curves were digitized using the IPDfromKM R package version 4.3.2. to reconstruct pseudo-IPD, from which HRs, RMSTs, and RMTLs were derived. Seven trials comprising 1,766 patients were included. The pooled HR for progression-free survival (PFS) was 0.67 [95% confidence interval (CI), 0.59–0.76] with an RMST gain of 1.84 months and RMTL reduction of 1.84 months. The pooled HR for overall survival (OS) was 0.73 (95% CI, 0.68–0.79) with an RMST gain of 1.98 months and RMTL reduction of 1.97 months. PH violations were more frequently observed in PFS than OS. Although HRs, RMSTs, and RMTLs were generally consistent, discrepancies in some trials underscore the value of RMST and RMTL as complementary, clinically intuitive measures. Incorporating RMST and RMTL into future ES-SCLC trials may improve the interpretability of treatment effects beyond conventional Cox model estimates.

**Significance:**

This first IPD meta-analysis evaluating RMST and RMTL as alternative endpoints in ES-SCLC demonstrates that these measures generally align with HR, with some exceptions. Given their clinical interpretability and ability to quantify survival benefit, RMST and RMTL should be considered as endpoints in future ES-SCLC trials.

## Introduction

Small cell lung cancer (SCLC) accounts for ≈13% of all lung cancers yet is responsible for a disproportionate share of lung cancer mortality owing to its aggressive biology, early metastatic spread, and near-universal relapse after first-line therapy (1). Around 70% of patients with SCLC are diagnosed with extensive-stage (ES) SCLC at the time of diagnosis. Chemo-immunotherapy is the current first-line treatment for patients with ES-SCLC, with modest survival benefits of only 2 to 3 months (2, 3).

As the field of cancer therapeutics evolves with the emergence of novel agents, it is essential to assess the actual efficacy of these new treatments to inform clinical practice and facilitate timely regulatory approvals. HR has served as the principal measure summarizing the effect size between two treatment groups for time-to-event outcomes in clinical trials. However, in the context of contemporary immune checkpoint inhibitors (ICI), survival curves may demonstrate delayed separations, long-lasting plateaus, or even crossovers, which can violate the fundamental assumptions of the Cox proportional hazards (PH) model underlying the HR and complicate the interpretation of HR, potentially obscuring the true clinical benefits (4). To address these limitations, alternative measures such as restricted mean survival time (RMST) and restricted mean time lost (RMTL) have been proposed, which present direct and absolute assessments of average survival time or time lost (5). These approaches are robust against nonproportional hazards (NPH) and offer a more intuitive representation of treatment benefit for patients. Considering the increasing significance of ICIs in the treatment of ES-SCLC, evaluating treatment impact through these metrics may provide a more accurate assessment of clinical benefits.

Therefore, we conducted a systematic review and meta-analysis of first-line ICI trials in ES-SCLC to evaluate the utility of RMST and RMTL as metrics to summarize treatment effects, comparing their insights with traditional HRs.

## Patients and Methods

### Data sources

MEDLINE, Embase, Cochrane, and ClinicalTrials.gov were searched to identify randomized phase III clinical trials in ES-SCLC in first- and second-line settings between 2014 and 2024. This analysis complied with the Preferred Reporting Items for Systematic Reviews and Meta-Analyses (PRISMA) Statement. Search terms included SCLC, randomized controlled trials (RCT), phase III, surrogate endpoint, overall survival (OS), progression-free survival (PFS), overall response rate, disease control rate, immunotherapy, checkpoint inhibitor, anti–PD-1, and anti–PD-L1. Additionally, we manually reviewed abstracts, posters, and presentations from major oncology conferences, including the American Society of Clinical Oncology, European Society for Medical Oncology, and International Association for the Study of Lung Cancer, including the World Conference on Lung Cancer. Reference lists from relevant systematic reviews and meta-analyses were also screened to identify any further eligible studies.

### Selection criteria

S. Durer and A. Dowlati independently screened the RCTs and extracted the following data: author, trial phase, sample size, treatment arms, line of therapy, and survival outcomes. Any discrepancies were resolved by consensus. Inclusion was limited to registered, randomized phase III clinical trials of first-line anti–PD-(L)1 for ES-SCLC, and studies were required to have available Kaplan–Meier curves for PFS and OS. Studies were required to report the NCT number. Phase I, II, nonrandomized, observational, retrospective, and maintenance therapy studies were excluded. We excluded trials using anti-CTLA and anti-TIGIT antibodies to maintain homogeneity for this meta-analysis as PD-(L)1 antibodies are more widely used. Protocols and ongoing studies without results, along with trials primarily focused on radiotherapy, were also excluded. Additionally, trials predominantly involving limited-stage SCLC were excluded.

### Assessment of risk of bias

The Cochrane Risk of Bias Tool was used for assessing the risk of bias in individual trials (6). The risks were scored as low, unclear, or high based on the random sequence generation, allocation concealment, blinding of participants and personnel, blinding of outcome assessment, incomplete outcome data, selective reporting, and other biases, which, respectively, indicated the assessment of selection bias, performance bias, detection bias, attrition bias, reporting bias, and other bias (Supplementary Fig. S1). Discrepancies were resolved by three adjudicators (S. Durer, P. Fu, and A. Dowlati).

### Data extraction and analysis

We extracted HRs for both OS and PFS, comparing patients treated with ICIs versus patients treated with standard of care (SOC) from randomized phase III trials that met the selection criteria. We used reported HRs and their 95% confidence intervals (CI) when available. If Kaplan–Meier curves with numbers at risk for both treatment modalities were provided, we used the graph digitizer software IPDfromKM R package version 4.3.2. (7) to extract coordinates of points on the curves and applied the numerical algorithm (7) to reconstruct survival results.

Using the same IPDfromKM R package, we extracted pseudo-individual patient data (IPD) from the Kaplan–Meier plots and estimated the RMST (8–10) per treatment group for each study and used the difference of RMST between treatment groups as another way to summarize the treatment effect on survival outcomes. RMST was estimated based on the smallest value (τ) among the largest observed times across the treatment groups. We also looked at the RMTL, which is defined as τ – RMST.

Weighted linear regression models investigated the association between log-HRs and difference of RMSTs (ΔRMSTs). The weighted linear regression (with weights equal to the inverse of variance of OR) takes the sample size of each study into consideration. As the precision (or inverse of variance) of estimate (e.g., OR) increases when the sample size increases, we put more weights for bigger studies. We used the logarithmic transformation on outcome measures (ORs and HRs) for variance stabilization, so the dependent variables of our regression models are approximately normally distributed

The heterogeneity of the studies selected for the meta-analysis was assessed with the tau^2^ and *χ*^2^-based Cochran Q test and quantified with the I^2^ (with values 25%, 25% to 75%, and ≥75% interpreted as representing low, moderate, and high levels of heterogeneity, respectively; ref. 11). The random effects model of DerSimonian and Laird with log (HR), RMST, and RMTL as dependent variables was utilized to pool studies and to correct the heterogeneity of the studies included for the meta-analysis (12). The effect size of treatment effect on time-to-event outcomes (OS and PFS) was reported as log (HR), SE, and HR with its 95% CI. The effect size of treatment on OS and PFS was also reported as the ΔRMSTs (ICI – SOC) or the difference of RMTLs (ICI – SOC). Publication bias was assessed using a funnel plot, which shows the relationship between the study SE and effect size, and with the Egger test (13). The *P* value of 0.05 was deemed statistically significant. The PH assumption of the Cox model was assessed using the Grambsch–Therneau test based on scaled Schoenfeld residuals (14). A violation of the PH assumption indicates that the HR between treatment groups changes over time rather than remaining constant as assumed by the Cox model. A *P* value less than 0.05 was considered evidence of violation of the PH assumption. When violations were observed, they were further explored visually using residual plots to assess the nature and extent of nonproportionality.

### Ethics statement

This study is a meta-analysis of previously published RCTs. All data used were obtained from publicly available sources, and no new patient data were collected. Therefore, institutional review board approval and informed consent were not required.

## Results

An initial search identified 2,257 publications for screening. After removing duplicates, 840 unique records remained and were subjected to title and abstract screening. Following this, 115 full-text articles were assessed for eligibility based on predefined inclusion and exclusion criteria. Ultimately, seven eligible studies (15–21), comprising a total of 1,766 patients, were included in this study. The PRISMA flow diagram details the study selection process at each stage (Supplementary Fig. S2). The main characteristics of the included trials are summarized in Supplementary Table S1. Most RCTs exhibited a low risk of bias across multiple domains.

The PH assumption was formally tested using a time-varying coefficient model. For PFS, a violation of the PH assumption was observed in four of seven studies: CASPIAN (*P* = 0.012; ref. 16), KEYNOTE-604 (*P* = 0.013; ref. 18), CAPSTONE-1 (*P* = 0.033; ref. 19), and EXTENTORCH (*P* = 0.003; ref. 20). The RATIONALE-312 study (21) showed a borderline violation (*P* = 0.072). For OS, PH violation was detected only in CAPSTONE-1 (*P* = 0.032, ref. 19), with RATIONALE-312 (21) again showing a borderline result (*P* = 0.099).

### Treatment effect estimates based on HR

**Figure 1:**
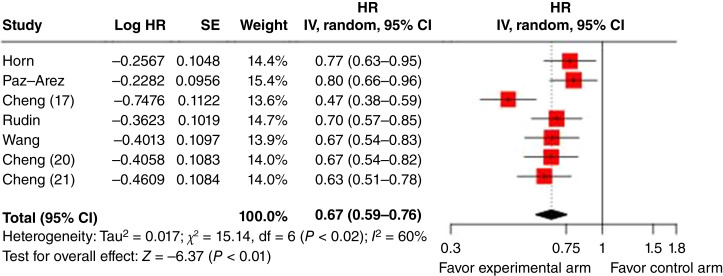
Forest plot of PFS HRs. The pooled estimate in log-scale, which was estimated using a mixed-effect model with log HR as the dependent variable, is −0.4 with SE of 0.063. IV, inverse variance method.

**Figure 2:**
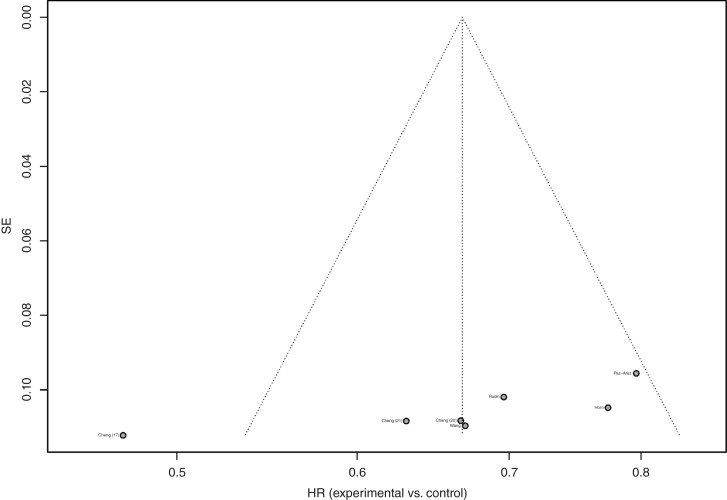
Funnel plot of PFS with *P* = 0.039 for testing funnel plot asymmetry.

The overall PFS results for individual studies and corresponding HR are shown in Fig. 1. The pooled HR for PFS was 0.67 (95% CI, 0.59–0.76; *P* < 0.0001). Moderate heterogeneity was observed among the trials (*χ*^2^ = 15.14; df = 6; *P* = 0.02; and *I*^2^ = 60%). The funnel plot analysis for PFS demonstrated significant asymmetry (*P* = 0.039) as shown in Fig. 2. The ASTRUM-005 study (17) reported the most pronounced treatment effect with a relatively higher SE than other studies. The magnitude of the effect size likely contributed to the observed asymmetry in the funnel plot.

**Figure 3:**
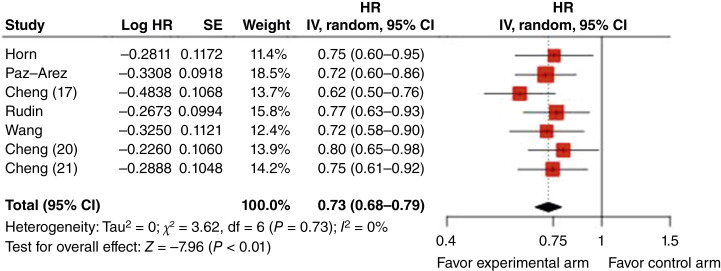
Forest plot of OS HRs. The pooled estimate in log-scale, which was estimated using a mixed-effect model with log HR as the dependent variable, is −0.315 with SE of 0.039. IV, inverse variance method.

**Figure 4:**
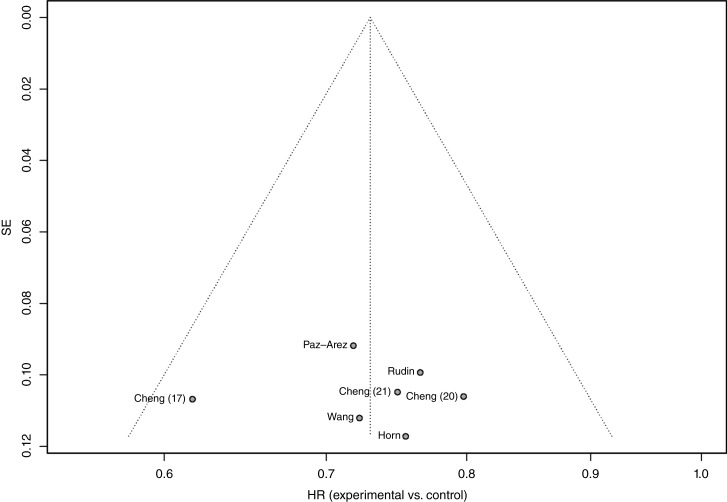
Funnel plot of OS with *P* = 0.986 for testing funnel plot asymmetry.

The overall OS results and corresponding HRs are shown in Fig. 3. The pooled HR for OS across studies was 0.73 (95% CI, 0.68–0.79; *P* < 0.0001). No heterogeneity was observed among the studies (*χ*^2^ = 3.62; df = 6; *P* = 0.73; and *I*^2^ = 0%). Unlike the PFS analysis, the funnel plot for OS showed no asymmetry (Fig. 4), likely because the treatment effect in ASTRUM-005 (17) was more aligned with the pooled estimate, despite a comparable SE. However, it is essential to acknowledge that the power of statistical tests for funnel plot asymmetry is limited when a small number of studies are included (*n* = 7).

### Treatment effect estimates based on RMST

**Figure 5:**
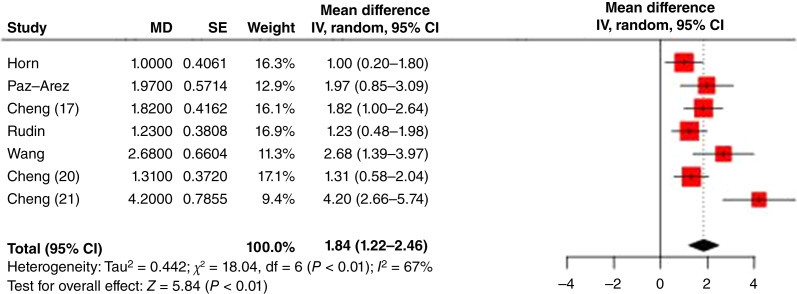
Forest plot of PFS RMSTs (MD = the difference of RMST between experimental and control). IV, inverse variance method.

The overall PFS results, including the difference in RMSTs for each study, are shown in Fig. 5. The pooled RMST gain in PFS across studies was 1.84 months (95% CI, 1.22–2.46). There was moderate heterogeneity in RMST (*χ*^2^ = 18.04; df = 6; *P* < 0.01; and *I*^2^ = 67%). The RATIONALE-312 study (21) demonstrated the largest RMST gain of 4.2 months despite having a PFS HR comparable with those of other studies. Another striking finding is that although the ASTRUM-005 study (17) demonstrated the most favorable HR for PFS, its RMST difference was consistent with those of other studies.

**Figure 6:**
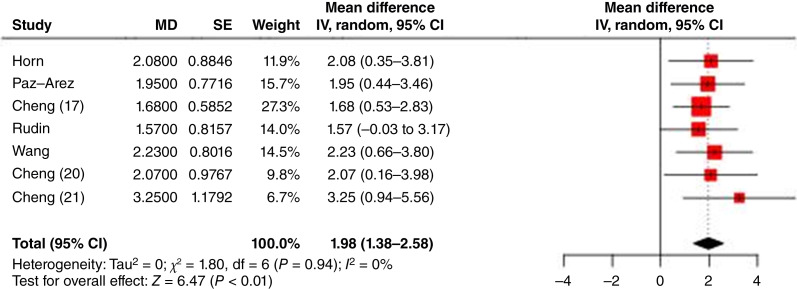
Forest plot of OS RMSTs (MD = the difference of RMST between experimental and control).IV, inverse variance method.

The overall OS results for the studies, along with the difference in RMSTs for each study, are shown in Fig. 6. The pooled RMST gain in OS across studies was 1.98 months (95% CI, 1.38–2.58). No evidence of heterogeneity was observed (*χ*^2^ = 1.8; df = 6; *P* = 0.94; and *I*^2^ = 0%). RMST (*χ*^2^ = 1.80) seems to be more robust with less heterogeneity than the Cox model (*χ*^2^ = 3.62) although neither test indicated statistically significant heterogeneity.

### Treatment effect estimates based on RMTL

**Figure 7:**
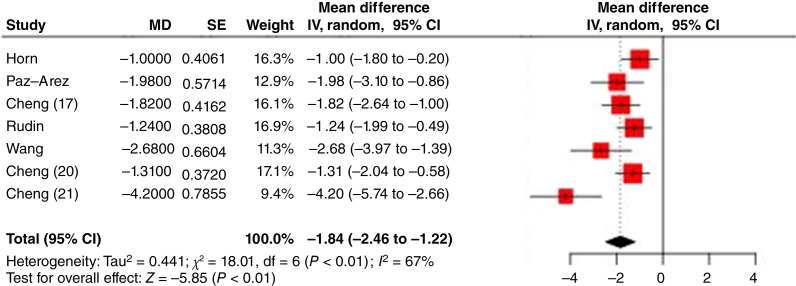
Forest plot of PFS RMTLs (MD = the difference of RMTL between experimental and control). IV, inverse variance method.

The overall PFS results for the studies, along with the differences in RMTLs for each study, are shown in Fig. 7. The pooled RMTL difference in PFS was −1.84 months (95% CI, −2.46 to −1.22). Moderate heterogeneity was observed across studies (*χ*^2^ = 18.01; df = 6; *P* < 0.01; and *I*^2^ = 67%). The RATIONALE-312 study (21) showed the greatest reduction in RMTL, indicating the most substantial benefit.

**Figure 8:**
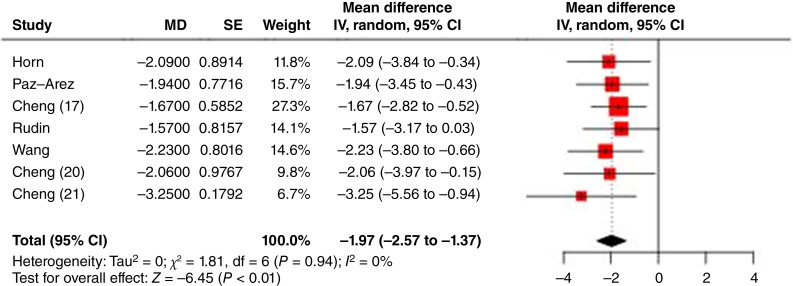
Forest plot of OS RMTLs (MD = the difference of RMTL between experimental and control). IV, inverse variance method.

The overall OS results for the studies, along with the difference in RMTLs for each study, are shown in Fig. 8. The pooled RMTL in OS was −1.97 months (95% CI, −2.57 to −1.37). Similarly, RATIONALE-312 (21) exhibited the lowest RMTL, consistent with the RMST findings.

### The association between log-HRs and ΔRMSTs

For PFS, there was no significant association between log-HRs and ΔRMSTs with a weighted Pearson correlation coefficient *r* = −0.15 (*P* = 0.741). For OS, however, a positive association was found between log-HRs and ΔRMSTs (*r* = 0.87; *P* = 0.012). These findings not only highlight the complementary nature of RMST and Cox modeling but also suggest that PFS may be a less reliable measure of long-term benefit, particularly in the setting of immunotherapy trials.

## Discussion

To our knowledge, this is the first IPD meta-analysis of first-line ICI trials of ES-SCLC investigating the value of RMST or RMTL as an endpoint in future trials. Our findings revealed that the pooled HRs for PFS and OS were 0.67 and 0.73, respectively. Correspondingly, RMST differences were 1.84 months for PFS and 1.98 months for OS, whereas the differences in RMTL were −1.84 months for PFS and −1.97 months for OS. Overall, the direction and significance of the differences in RMST and RMTL align with the HR, with some exceptions.

We observed a violation of the PH assumption in four of seven studies for PFS, and in one study for OS, indicating that HRs should be interpreted with caution. Among the included trials, ASTRUM-005 (17) demonstrated the most favorable HRs for both OS and PFS, yet the RMST gains were comparable with those in other studies, highlighting that pronounced treatment effects based on the Cox model do not always correspond to a greater absolute survival benefit. In contrast, RATIONALE-312 (21) showed the largest improvements in RMST and RMTL despite consistent results with other studies in the Cox model. This suggests that time-based metrics to summarize treatment effects may more accurately reflect extended survival benefits, particularly when survival curves diverge late.

Although formal testing in RATIONALE-312 (21) yielded a borderline PH violation (*P* = 0.099), visual inspection of the Kaplan–Meier curves revealed early crossover around 6 months, followed by late separation favoring tislelizumab. Despite a modest median OS improvement (15.5 vs. 13.5 months), the long-term survival advantage observed at the tail of the curve suggests a durable benefit that HR may underestimate. Therefore, incorporating alternative measures such as RMST or RMTL may provide a more robust assessment.

Additionally, we observed heterogeneity in PFS when using both the Cox model and RMST analyses, whereas no heterogeneity was evident for OS across either metric. This discrepancy likely reflects the greater sensitivity of PFS to trial-specific factors, such as variations in disease assessment methods, imaging intervals, and reader interpretation, compared with OS. Unlike PFS, OS is a well-defined and objective endpoint, which likely explains the consistency observed across trials. PFS and OS remain the most widely used time-to-event endpoints. PFS is valued for providing earlier signals of disease control and treatment activity, which can accelerate trial readouts and regulatory decisions. However, its limitations include dependence on assessment frequency and its inability to capture quality of life. By contrast, OS directly reflects treatment benefit by quantifying survival extension and serves as the benchmark for establishing new SOCs. However, it requires longer follow-up and may be confounded by crossover and post-progression therapies. RMST can be applied irrespective of whether the PH assumption is met; it provides a model-free, absolute measure of treatment effect. Studies suggest that RMST is similar to HR in identifying differences between groups when the hazards are proportional, but it performs better when the hazards are not proportional. The agreement between HR- and RMST-based estimates strengthens the observed survival advantage, showing that RMST enhances interpretability rather than serving as a fallback when the PH assumption is violated.

A systematic review of 54 phase III oncology trials involving more than 33,000 patients across various cancer types and treatment modalities found that HRs produced larger treatment effect estimates than RMST-based measures (10). HRs frequently seemed more favorable when the absolute survival gain was small, and this finding was consistent regardless of the PH assumption. These results align with our observations, in which pronounced HR effects (e.g., ASTRUM-005) did not translate into proportionately greater RMST gains, whereas RMST more effectively captured late survival separation as seen in RATIONALE-312. A subsequent meta-analysis on 25 RCTs evaluating ICIs found evidence of NPH in 28% of included trials (22). Their analysis also revealed that HRs consistently provided larger estimates of treatment effect than the ratio of RMST or RMTL across all included ICI trials. Although HR- and RMST-based measures generally agreed on the direction and statistical significance of effect, discordance was observed in some trials. Both studies, therefore, collectively underscore that the limitations of HRs and the advantages of RMST/RMTL are not confined to a specific treatment class or type of cancer.

A comparative analysis revisiting the CheckMate 057 trial, which evaluated nivolumab versus docetaxel in advanced non-squamous non–small cell lung cancer, highlighted the limitations of HR under NPH (4). The trial failed to demonstrate a significant improvement in PFS (HR, 0.92; *P* = 0.39), and the PFS curves crossed, violating the PH assumption. RMST analysis at 24 months demonstrated statistically significant benefits in PFS (+1.3 months; 95% CI, 0.3–2.3; *P* = 0.02) in favor of nivolumab. In this context, the RMST-based procedure proved to be a more powerful tool for detecting treatment effects. Moreover, the analysis of the dynamic RMST curve for OS for CheckMate 057 revealed an initial period (before approximately 10 months) during which the control arm performed better, followed by an improvement in the treatment arm’s benefit, eventually reaching a plateau at which no clear long-term difference between treatments was observed (23). This detailed characterization of time-varying effects, including crossing survival curves and transient or diminishing benefits, unequivocally underscores the superior ability of RMST to capture complex survival patterns, providing valuable information that is not readily obtainable through the standard HR approach.

Along with the difference in or the ratio of RMST, RMTL may also be a useful summary measure. It is currently unknown which is preferred between RMST and RMTL although some studies of their application to IPD from published clinical trials have reported that they have similar properties (24). The ExteNET trial, which evaluated adjuvant neratinib in early-stage HER2-positive breast cancer, offers a compelling example of the key considerations involved in interpreting survival endpoints (25). Although the HR was 0.67, indicating a substantial reduction in hazard, the corresponding absolute gain in RMST was 0.5 months, raising questions about the clinical significance of a statistically robust finding, a common dilemma in oncology. Notably, in this low event-rate adjuvant setting, the RMTL ratio was approximately 0.5, closely aligning with the HR. Such examples underscore the value of RMST and RMTL as complementary metrics to HR, capable of providing additional insights into treatment effects that HR alone may not fully capture. Moreover, the HR from the Cox model is complex to translate at the bedside. Expressing the treatment effect in terms of additional months of life and months gained makes the benefit concrete for patients and clinicians. These absolute gains complement the HR, remain informative when hazards are not strictly proportional, and help with shared decision-making in a disease in which baseline survival is short.

When designing a clinical trial using RMST as an endpoint, the sample size should be calculated based on the desired power and significance level. Royston and Parmar have extensively studied statistical considerations for such designs (8). They demonstrated that an analysis combining both RMST and PH tests can be conducted with only a modest increase in sample size, typically less than 10% more than that required for a PH-based test alone. Key limitations of RMST and RMTL include the dependence on the prespecified restriction time (τ) although dynamic RMST analysis has been proposed in the literature (23), potential instability or bias under limited follow-up or heavy/differential right-censoring, reduced statistical efficiency compared with HRs under Cox proportional hazards model, challenges in cross-trial comparisons due to varying τ, and the evolving clinical and regulatory familiarity with these measures.

Our study has several limitations. First, although we limited the analysis to trials evaluating anti–PD-(L)1 inhibitors to reduce heterogeneity, variations in study design, patient populations, and control regimens remain. Second, we did not have access to true IPD, which restricted our ability to assess patient-level data. Instead, we utilized reconstructed IPD derived from published Kaplan*–*Meier curves. Prior validation studies have shown that RMST estimates derived from reconstructed IPD displayed excellent accuracy and low predictive error compared with the gold-standard RMST estimates calculated using the original trial IPD (Louis Everest). Reconstructed IPD has limitations as it cannot fully capture potential digitization and reconstruction errors, relies on reported numbers at risk and assumptions of noninformative censoring, and lacks access to patient-level covariates, stratification, and details on subsequent therapies, making it challenging to evaluate informative censoring while also being affected by instability in the tails when follow-up is immature, and depending on the quality of reporting in Kaplan–Meier plots and at-risk tables. Third, despite efforts to assess and address potential bias, the inherent risk of small-study effects remains.

In conclusion, this meta-analysis demonstrates that RMST and RMTL serve as valuable complementary metrics to HR for assessing the treatment effect of ICIs in ES-SCLC. Although overall benefits were generally consistent across HR, RMST, and RMTL analyses, notable discrepancies were observed in some trials. Nevertheless, RMST clarified survival gains even in trials characterized by NPH. As the role of immunotherapy continues to expand, integrating RMST or RMTL into future trial designs is crucial for providing clearer and absolute insights into treatment impact, particularly when relative effects may seem disproportionately large.

## Supplementary Material

Supplementary Figure 1 Cochrane risk of bias tool

Supplementary Figure 2 The Prisma Flow Diagram.

Supplementary Table 1 Characteristics of included studies

## Data Availability

The IPD used in this study were reconstructed from published Kaplan–Meier curves using the IPDfromKM R package. Data and programs (R and SAS) are available from the GitHub repository at https://github.com/pxf16/RMST-as-an-alternative-approach-to-evaluate-effect-size-of-treatment-on-survival-in-ES-SCLC.

## References

[1] Rudin CM , Brambilla E, Faivre-Finn C, Sage J. Small-cell lung cancer. Nat Rev Dis Primers 2021;7:3. 33446664 10.1038/s41572-020-00235-0 PMC8177722

[2] Paz-Ares L , Dvorkin M, Chen Y, Reinmuth N, Hotta K, Trukhin D, . Durvalumab plus platinum-etoposide versus platinum-etoposide in first-line treatment of extensive-stage small-cell lung cancer (CASPIAN): a randomised, controlled, open-label, phase 3 trial. Lancet 2019;394:1929–39. 31590988 10.1016/S0140-6736(19)32222-6

[3] Horn L , Mansfield AS, Szczęsna A, Havel L, Krzakowski M, Hochmair MJ, . First-line atezolizumab plus chemotherapy in extensive-stage small-cell lung cancer. N Engl J Med 2018;379:2220–9. 30280641 10.1056/NEJMoa1809064

[4] Pak K , Uno H, Kim DH, Tian L, Kane RC, Takeuchi M, . Interpretability of cancer clinical trial results using restricted mean survival time as an alternative to the hazard ratio. JAMA Oncol 2017;3:1692–6. 28975263 10.1001/jamaoncol.2017.2797 PMC5824272

[5] A’Hern RP . Restricted mean survival time: an obligatory end point for time-to-event analysis in cancer trials? J Clin Oncol 2016;34:3474–6. 27507871 10.1200/JCO.2016.67.8045

[6] Cumpston M , Li TJ, Page MJ, Chandler J, Welch VA, Higgins JPT, . Updated guidance for trusted systematic reviews: a new edition of the Cochrane Handbook for Systematic Reviews of Interventions. Cochrane Db Syst Rev 2019;10:ED000142. 10.1002/14651858.ED000142 PMC10284251 31643080

[7] Liu N , Zhou Y, Lee JJ. IPDfromKM: reconstruct individual patient data from published Kaplan-Meier survival curves. BMC Med Res Methodol 2021;21:111. 34074267 10.1186/s12874-021-01308-8 PMC8168323

[8] Royston P , Parmar MK. Restricted mean survival time: an alternative to the hazard ratio for the design and analysis of randomized trials with a time-to-event outcome. BMC Med Res Methodol 2013;13:152. 24314264 10.1186/1471-2288-13-152 PMC3922847

[9] Uno H , Claggett B, Tian L, Inoue E, Gallo P, Miyata T, . Moving beyond the hazard ratio in quantifying the between-group difference in survival analysis. J Clin Oncol 2014;32:2380–5. 24982461 10.1200/JCO.2014.55.2208 PMC4105489

[10] Trinquart L , Jacot J, Conner SC, Porcher R. Comparison of treatment effects measured by the hazard ratio and by the ratio of restricted mean survival times in oncology randomized controlled trials. J Clin Oncol 2016;34:1813–9. 26884584 10.1200/JCO.2015.64.2488

[11] Higgins JP , Thompson SG, Deeks JJ, Altman DG. Measuring inconsistency in meta-analyses. BMJ 2003;327:557–60. 12958120 10.1136/bmj.327.7414.557 PMC192859

[12] DerSimonian R , Laird N. Meta-analysis in clinical trials revisited. Contemp Clin Trials 2015;45:139–45. 26343745 10.1016/j.cct.2015.09.002 PMC4639420

[13] Egger M , Davey Smith G, Schneider M, Minder C. Bias in meta-analysis detected by a simple, graphical test. BMJ 1997;315:629–34. 9310563 10.1136/bmj.315.7109.629 PMC2127453

[14] Grambsch PM , Therneau TM. Proportional hazards tests and diagnostics based on weighted residuals. Biometrika 1994;81:515–26.

[15] Liu SV , Reck M, Mansfield AS, Mok T, Scherpereel A, Reinmuth N, . Updated overall survival and PD-L1 subgroup analysis of patients with extensive-stage small-cell lung cancer treated with atezolizumab, carboplatin, and etoposide (IMpower133). J Clin Oncol 2021;39:619–30. 33439693 10.1200/JCO.20.01055 PMC8078320

[16] Paz-Ares L , Chen Y, Reinmuth N, Hotta K, Trukhin D, Statsenko G, . Durvalumab, with or without tremelimumab, plus platinum-etoposide in first-line treatment of extensive-stage small-cell lung cancer: 3-year overall survival update from CASPIAN. ESMO Open 2022;7:100408. 35279527 10.1016/j.esmoop.2022.100408 PMC9161394

[17] Cheng Y , Han L, Wu L, Chen J, Sun H, Wen G, . Serplulimab vs. placebo combined with chemotherapy as first-line treatment for extensive-stage small-cell lung cancer: extended follow-up results and patient-reported outcomes from the international phase 3 ASTRUM-005 study. J Clin Oncol 2024;42(Suppl 16):8100.

[18] Rudin CM , Awad MM, Navarro A, Gottfried M, Peters S, Csőszi T, . Pembrolizumab or placebo plus etoposide and platinum as first-line therapy for extensive-stage small-cell lung cancer: randomized, double-blind, phase III KEYNOTE-604 study. J Clin Oncol 2020;38:2369–79. 32468956 10.1200/JCO.20.00793 PMC7474472

[19] Wang J , Zhou C, Yao W, Wang Q, Min X, Chen G, . Adebrelimab or placebo plus carboplatin and etoposide as first-line treatment for extensive-stage small-cell lung cancer (CAPSTONE-1): a multicentre, randomised, double-blind, placebo-controlled, phase 3 trial. Lancet Oncol 2022;23:739–47. 35576956 10.1016/S1470-2045(22)00224-8

[20] Cheng Y , Zhang W, Wu L, Zhou C, Wang D, Xia B, . Toripalimab plus chemotherapy as a first-line therapy for extensive-stage small cell lung cancer: the phase 3 EXTENTORCH randomized clinical trial. JAMA Oncol 2024;11:16–25. 10.1001/jamaoncol.2024.5019 PMC11565370 39541202

[21] Cheng Y , Fan Y, Zhao Y, Huang D, Li X, Zhang P, . Tislelizumab plus platinum and etoposide versus placebo plus platinum and etoposide as first-line treatment for extensive-stage SCLC (RATIONALE-312): a multicenter, double-blind, placebo-controlled, randomized, phase 3 clinical trial. J Thorac Oncol 2024;19:1073–85. 38460751 10.1016/j.jtho.2024.03.008

[22] Liang F , Zhang S, Wang Q, Li W. Treatment effects measured by restricted mean survival time in trials of immune checkpoint inhibitors for cancer. Ann Oncol 2018;29:1320–4. 29788167 10.1093/annonc/mdy075

[23] Liao JJZ , Liu GF, Wu W-C. Dynamic RMST curves for survival analysis in clinical trials. BMC Med Res Methodol 2020;20:218. 32854619 10.1186/s12874-020-01098-5 PMC7534804

[24] Han K , Jung I. Restricted mean survival time for survival analysis: a quick guide for clinical researchers. Korean J Radiol 2022;23:495–9. 35506526 10.3348/kjr.2022.0061 PMC9081686

[25] Martin M , Holmes FA, Ejlertsen B, Delaloge S, Moy B, Iwata H, . Neratinib after trastuzumab-based adjuvant therapy in HER2-positive breast cancer (ExteNET): 5-year analysis of a randomised, double-blind, placebo-controlled, phase 3 trial. Lancet Oncol 2017;18:1688–700. 29146401 10.1016/S1470-2045(17)30717-9

