# Supplementary material for: Roles of Restricted Mean Survival Time and Restricted Mean Time Lost in Evaluating Immune Checkpoint Inhibitor Efficacy for Extensive-Stage Small Cell Lung Cancer
Source: Cancer Res Commun. 2026 Jan 12;6(1):77–84. doi: 10.1158/2767-9764.CRC-25-0387 (PMC12791115; doi:10.1158/2767-9764.CRC-25-0387)
Supplement: Supplementary Figure 1 — Cochrane risk of bias tool [file crc-25-0387_supplementary_figure_1_suppsf1.docx]

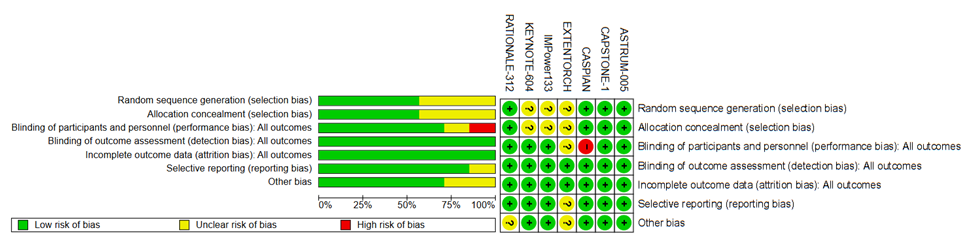


**Supplementary Figure 1:** Summary of results from assessment of studies using the Cochrane risk of bias tool. Across the seven RCTs, most domains were low risk. Allocation concealment was unclear in three studies. Performance bias and detection bias were high in only one study
