# Supplementary material for: Roles of Restricted Mean Survival Time and Restricted Mean Time Lost in Evaluating Immune Checkpoint Inhibitor Efficacy for Extensive-Stage Small Cell Lung Cancer
Source: Cancer Res Commun. 2026 Jan 12;6(1):77–84. doi: 10.1158/2767-9764.CRC-25-0387 (PMC12791115; doi:10.1158/2767-9764.CRC-25-0387)
Supplement: Supplementary Figure 2 — The Prisma Flow Diagram. [file crc-25-0387_supplementary_figure_2_suppsf2.docx]

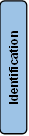

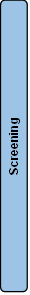

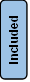


Records identified through database searching (n=2257)

Pubmed: 172

Cochrane: 539

Embase: 1096

Clinicaltrials.gov: 450

Records after duplicates removed (n=840)

Reports assessed for eligibility

(n = 115)

**Supplementary Figure 2:** The PRISMA flow diagram

Duplicates removed (n=1417)

Records excluded by titles/abstracts (n =725)

-Incorrect patient population or stage (n=165)

-Wrong line/intent (maintenance, consolidation, ≥2nd-line) (n=120)

-Nontherapeutic/prognostic/biomarker-only (n=140)

-Preclinical studies (n=112)

-Radiation-only or surgery-only interventions (n=88)

-Review, editorial, commentary (n=85)

-Insufficient data (n=15)

Full text articles excluded (n=91)

-Only protocol without results (n=14)

-Phase I, II, IIb, IIIb studies (n=50)

-Mainly radiation studies (n=27)

-Phase III studies without immunotherapy and immunotherapy targeting CTLA4 (n=17)

Studies included

(n = 7)
