# Supplementary material for: Roles of Restricted Mean Survival Time and Restricted Mean Time Lost in Evaluating Immune Checkpoint Inhibitor Efficacy for Extensive-Stage Small Cell Lung Cancer
Source: Cancer Res Commun. 2026 Jan 12;6(1):77–84. doi: 10.1158/2767-9764.CRC-25-0387 (PMC12791115; doi:10.1158/2767-9764.CRC-25-0387)
Supplement: Supplementary Table 1 — Characteristics of included studies [file crc-25-0387_supplementary_table_1_suppst1.docx]

**Supplementary Table 1:** Characteristics of included phase III studies

| **Author** | **Year** | **PMID** | **Trial name** | **Target** | **Experimental arm** | | **Control arm** | | **ORR** | | **mPFS (months) (95% CI)** | | | **mOS (months) (95% CI)** | | |
| --- | --- | --- | --- | --- | --- | --- | --- | --- | --- | --- | --- | --- | --- | --- | --- | --- |
|  |  |  |  |  | **treatment** | ***N*** | **Control** | ***N*** | ***treatment*** | ***control*** | ***treatment arm*** | ***control arm*** | ***HR*** | ***treatment arm*** | ***control arm*** | ***HR*** |
| Horn  (14) | 2018/  2020 | 30280641 | IMPower133 | PD-L1 | Atezo+EP | 201 | EP | 202 | 60.2% | 64.40% | 5.2  (4.4-5.6) | 4.3  (4.2-4.5) | 0.77  (0.63-0.95) | 12.3  (10.8-15.8) | 10.3  (9.3-11.3) | 0.76  (0.6-0.95) |
| Paz-Arez(2)  (15) | 2019/  2022 | 35279527 | CASPIAN | PD-L1 | Durva+EP | 268 | EP | 269 | 68% | 58% | 5.1  (4.7-6.2) | 5.4  (4.8-6.2) | 0.8  (0.66-0.96) | 12.9  (11.3-14.7) | 10.5  (9.3-11.2) | 0.71  (0.6-0.86) |
| Cheng Y  (16) | 2022 | 36166026 | ASTRUM-005 | PD-1 | Serplulimab+  EP | 389 | EP | 196 | 80% | 70% | 5.7  (5.5-6.9) | 4.3  (4.2-4.5) | 0.48  (0.38-0.59) | 15.4  (13.3-NE) | 10.9  (10-14.3) | 0.63  (0.49-0.82) |
| Rudin  (17) | 2022 | 32468956 | KEYNOTE-604 | PD-1 | Pembro+EP | 228 | EP | 225 | 71% | 61.8% | 4.8  (4.3-5.4) | 4.3  (4.2-4.5) | 0.7  (0.57-0.85) | 10.8  (9.2-12.9) | 9.7  (8.6-10.7) | 0.76  (0.63-0.93) |
| Wang  (18) | 2022 | 35576956 | CAPSTONE-1 | PD-L1 | Adebrelimab+EP | 230 | EP | 232 | 70% | 66% | 5.8  (5.6-6.9) | 5.6  (5.5-5.7) | 0.67  (0.54-0.83) | 15.3  (13.2-17.5) | 12.8  (11.3-13.7) | 0.73  (0.58-0.9) |
| Cheng Y  (19) | 2023 | 39541202 | EXTENTORCH | PD-1 | Toripalimab+  EP | 223 | EP | 219 | NA | NA | 5.8 | 5.6 | 0.67  (0.539-0.824) | 14.6 | 13.3 | 0.79  (0.648-0.982) |
| Cheng Y  (20) | 2023 | 38460751 | RATIONALE-312 | PD-1 | Tislelizumab+EP | 227 | EP | 230 | 68.3% | 61.70% | 4.8 | 4.3 | 0.63  (0.51-0.78) | 15.5 | 13.5 | 0.75  (0.61-0.92) |

**Abbreviations:** Atezo: Atezolizumab; EP: Etoposide+Platinum; HR: Hazard ratio; mPFS: Median progression free survival; mOS: Median overall survival; ORR: Objective response rate
